# Evaluating the Anterior Loop of the Mental Nerve Using Cone Beam CT Scans in the Jordanian Population

**DOI:** 10.7759/cureus.58519

**Published:** 2024-04-18

**Authors:** Ahmed Al dalalah, Nadeem Kana’an, Ala’ Ersheidat, Moath Momani, Huthaifa Altantawi

**Affiliations:** 1 Department of Restorative Dentistry, Royal Medical Services, Amman, JOR; 2 Department of Prosthodontics, Royal Medical Services, Amman, JOR; 3 Department of Periodontics, Royal Medical Services, Amman, JOR

**Keywords:** cone beam computed tomography, mental foramen (mf), dental landmarks, mental nerve (mn), anterior loop

## Abstract

Introduction: Knowledge of anatomical landmarks is crucial for effective dental treatments, especially in surgical procedures. The mental nerve (MN), a branch of the inferior alveolar nerve, features a critical landmark known as the anterior loop (AL), often overlooked by surgeons. This study aims to assess the occurrence of the MN AL, its type, and its length within a sample of the Jordanian population by utilizing cone beam computed tomography (CBCT) scans.

Methods: This retrospective observational study included the acquisition of CBCT images from a total of randomly selected 268 patients who sought treatment for a range of dental conditions (such as tooth extraction, orthodontic therapy, and dental implants) at hospitals affiliated with the Jordanian Royal Medical Services. Reformatted images were utilized to detect the AL type, length, and the association between the nerve type and mental foramen (MF) position.

Results: This study involved 268 patients. The distribution of MF positions and the characteristics of the inferior dental nerve were evaluated, with no significant gender differences observed. The predominant location for the MF in both males and females in both sides was position IV, with 52% of females and 56-59% (left-right sides) of males presenting this trait. The inferior dental nerve types also showed no significant gender variation, with 42-43% (left-right sides) of females having type III and a similar distribution among males. Measurements of the midline-mental foramen and inter-foramen distances revealed slight variations between genders, with significant differences noted in the right AL length for type III nerves, favoring males (p=0.034). A notable correlation was found between the type of inferior dental nerve and the MF position, particularly with type I nerves predominantly associating with position IV mental foramina (p≤0.004).

Conclusion: CBCT scans are essential in the precise evaluation of the AL, aiding in the prevention of nerve injuries during dental procedures. Our results highlight the diversity of the AL in the Jordanian population and the importance of individualized treatment plans. Future research with larger cohorts is advised to refine these insights, aiming to improve treatment outcomes and patient care.

## Introduction

The mental nerve (MN) represents a terminal branch of the inferior alveolar nerve, emerging through the mental foramen (MF) to provide sensory innervation to the skin and mucous membrane of the buccal vestibule in the mandible. Its distribution spans from the medial edge of the masseter muscle to the midline [1]. The MN provides innervation to areas like the lower lip, chin, and mucous membranes of both. It plays a role in our ability to perceive touch, pain, and temperature in these regions of our face. Consequently, any injury or damage to this nerve can lead to complications such as sensation, pain, or numbness in areas innervated by the nerve.

Understanding MF positions is crucial for the performance of anesthesia on the MN or the avoidance of nerve damage during surgical procedures in the premolar area of the mandible [2]. Tebo and Telford classified the location of the MF relative to the mandibular teeth into six positions [3]. These positions are categorized as follows: position I, found mesial to the first premolar; position II, aligned with the long axes of the first premolar; position III, situated between the first premolar and second premolar; position IV, aligned with the long axes of the second premolar; position V, located between the second premolar and the first molar; and position VI, positioned at the mesial half of the first molar. In dental operations, knowledge of MF positions is crucial since they are key anatomical considerations to take into account during implant placement or performing surgical procedures in the mandibular premolar region.

The anterior loop (AL) is a continuation of the inferior alveolar nerve that extends forward, beyond the mental foramen, which loops back to exit the MF. The significance of the AL inside the mandible should not be underestimated, as its proper evaluation is crucial to preventing any potential damage during surgical procedures. The AL may be classified into three distinct categories, namely, Type I, Type II, and Type III. Type I demonstrates an anatomical structure that resembles the letter Y, while Type II presents an anatomical structure like the letter T. In contrast, Type III reveals an architectural arrangement resembling the letter Y, with the incisive branch being thicker than the main branch [4].

Understanding the structure and function of the MN is crucial in dentistry, particularly the AL when determining a safe interforaminal area for procedures such as dental implants, open reduction of mandibular fractures, genioplasty, tooth extractions, and endodontic treatments to prevent neurosensory disturbances.

With the increase in awareness about dental care and its positive impact on the health of an individual, there has been a significant boost in the research field for the newer technologies to be used in the dental sector [5]. The introduction of newer technologies has significantly increased the quality of treatment being provided to individuals. One of the technologies for the dental sector that has been very crucial is “cone beam computed tomography” (CBCT) [6]. This technology enables us to get a very detailed image of the mouth and face structures. By using CBCT scans and carefully examining AL, experts can detect specific difficulties or differences that are specific to the Jordanian population. This understanding enables the creation of personalized treatment strategies that reduce risks and increase the effectiveness of the treatments.

There is a notable gap in the literature regarding the Jordanian population's specific dental anatomical features and their treatment implications. Thus, our study aims to assess the mental nerve anterior loop using CBCT scans and provide insights into its prevalence, anatomical variations, and length to enhance dental treatment planning, outcomes, and reduce the incidence of neurosensory complications. 

## Materials and methods

This is a retrospective study on 268 CBCT images of patients' records from the period 1/2023 to 7/2023. The patients were randomly selected from those seeking various dental treatments at facilities under the Jordanian Royal Medical Services. The cohort comprised 134 females and 134 males, aged between 18 and 75 years, who met specific inclusion and exclusion criteria to ensure a homogenous study population. The Institutional Review Board (IRB) at the Royal Medical Services-Jordan (Number 20.8/2023) approved the study. The study was performed in accordance with the principles of the Declaration of Helsinki, 1975. Informed consent was waived by the IRB committee due to the retrospective nature of the study. Patient data was anonymized and maintained with complete confidentiality.

The inclusion criteria encompassed individuals aged 18 to 75 from both genders who had undergone previous X-ray imaging for measurements related to the foramen. Participants were required to provide informed consent and possess a complete set of teeth without any significant pathologies affecting the mandibular region. Only images free of artifacts were included in the analysis. Conversely, the exclusion criteria ruled out individuals with lesions (either radiolucent or radio-opaque) in the foramen area, any anomalies or pathologies like cysts or tumors affecting the region, and those with supernumerary or unerupted teeth that obstructed the foramen visualization. For subjects with radiographs demonstrating exposure to or processing artifacts, known developmental abnormalities affecting the foramen, or a history of trauma or surgical interventions, we relied on the Kodak 9500 Cone Beam 3D System (Carestream Health, Rochester, USA) following a protocol and scan parameters of 90 kVp and 10 mA [7]. The exposure time was 12 s, the effective exposure time was 2-5 s, and the voxel size was 0.2 mm × 0.2 mm × 0.2 mm. Trained operators were present during the CBCT scans to ensure consistency and adherence to established guidelines [8].

The DICOM 3D visualization software allowed us to thoroughly evaluate the parameters of the foramen [9]. With the help of CBCT technology, we obtained high-resolution images that facilitated measurements and accurate assessments of the MF and AL in our study participants [10]. The photographs obtained were subjected to meticulous analysis by two dentists, each with a minimum of eight years of professional experience. In instances where discrepancies arose in the evaluations made by the two observers, a consensus was achieved through thorough discussion.

**Figure 1:**
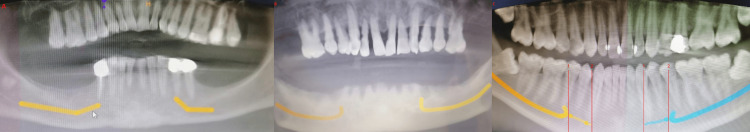
Classification of the anterior loop of the mental nerve. (A) Type I; (B) Type II; (C) Type III (1: Mental foramen, 2: End of loop)

We used the method described by Sahman et al. to evaluate the CBCT images, using the sagittal, cross-sectional, and multiplanar reformatted images of the CBCT scans [11]. This approach allowed us to classify the type of the anterior loop and for measurement of its length. For loop type, we categorized them into Type I, Type II, and Type III, as shown in Figure 1, based on the anatomical configuration and branching patterns of the MN as it traverses the MF. As for the length, it was precisely measured from the most anterior point of the loop to the MN. Other measurements included the midline-MF distance, the inter foramen distance, and the length of the AL for type III.

To assess the agreement between examiners regarding the linear parameters such as the midline mental foramen distance, interforamen distance, and length of the anterior loop of Type II, the intraclass correlation coefficient was employed, with values below 0.5 suggesting poor reliability, while those falling between 0.5 and 0.75 signify moderate reliability. Values ranging from 0.75 to 0.9 suggest good reliability, and those above 0.90 indicate excellent reliability.

Categorical variables were described as counts and percentages (%), and continuous variables were described as mean (standard deviation (SD)) if the data was normally distributed based on the Shapiro-Wilk test, or median (range) if data deviated from normality. To compare continuous variables, the T-test or Mann-Whitney U test was utilized, for categorical variables, the Chi-Squared test was used if the category count was >5 otherwise, Fisher's exact test was utilized. IBM SPSS Statistics for Windows, Version 29 (Released 2023; IBM Corp., Armonk, New York, United States) was used to carry out the statistical analysis [12].

## Results

The investigators showed almost perfect reproducibility with high intraclass correlation coefficient values for the left and right midline mental foramen distance (90%), interforamen distance (97%), length of the right anterior loop for Type III (82%), and length of the left anterior loop for Type III (81%) as shown in Table 1. 

**Table 1:** Intraclass correlation coefficient for inter-rater reliability for the study parameter.

Variable	Intraclass correlation coefficient	95% CI	p-value
Midline Mental Foramen Distance (Right)	90%	0.87-0.92	< .001
Midline Mental Foramen Distance (Left)	90%	0.87-0.92	< .001
Interforamen Distance	97%	0.97-0.98	< .001
Length of Anterior Loop for Type III (Right)	82%	0.78-0.86	< .001
Length of Anterior Loop for Type III (Left)	81%	0.77-0.85	< .001

A total of 268 patients were included, with 134 (50%) being females and 134 (50%) being males. The most common MF is in position IV, observed in 52% of females and 56-59% of males on both sides, with no significant difference between females and males in the MF position on both sides, as shown in Table 2. The inferior dental nerve type varied among females and males with no significant difference, in which 56 (42%) of females had type III inferior dental nerve on the right side, and 49 (37%) had type III on the left side, while 58 (43%) of males had type I inferior dental nerve on the right side and 56 (42%) had type III on the left side. The median (IQR) right midline-mental foramen distance was 27.0 (44.3, 49.2) in females, and 27.1 (26.0, 28.8) in males, while the median left midline-mental foramen distance was 26.5 (25.4, 28.4) in females and 27.4 (25.5, 28.9) in males. Median inter-foramen distances ranged from 46.8 (44.3, 49.2) to 47.2 (45.0, 49.3) mm for both females and males, showing no significant variation between genders. The median right length of the AL for type III differed significantly between females and males, in which females had a median of 2.40 (1.83, 3.0) and 2.90 (2.0, 3.90) in males (p-value = 0.034), while the median length of the left AL for type III did not differ significantly between males and females.

**Table 2:** Patients’ characteristics and differences based on gender. ^1^n (%); Median (IQR) ^2^Fisher’s exact test; Wilcoxon rank sum test; Pearson’s Chi-squared test

Characteristic	Right-side			Left-side		
	Females (N = 134^1^)	Males (N = 134^1^)	p-value^2^	Females (N = 134^1^)​​​​​​​	Males (N = 134^1^)	p-value^2^
Mental foramen position						
I	1 (0.8%)	0 (0%)	0.9			0.5
II	2 (1.5%)	2 (1.5%)		3 (2.3%)	1 (0.8%)	
III	49 (37%)	43 (33%)		51 (38%)	42 (32%)	
IV	69 (52%)	74 (56%)		69 (52%)	78 (59%)	
V	12 (9.0%)	13 (9.8%)		10 (7.5%)	11 (8.3%)	
Unknown	1	2		1	2	
ID nerve type			0.14			0.2
I	49 (37%)	58 (43%)		50 (37%)	55 (41%)	
II	29 (22%)	17 (13%)		35 (26%)	23 (17%)	
III	56 (42%)	59 (44%)		49 (37%)	56 (42%)	
Midline-mental foramen distance	27.0 (25.5, 28.6)	27.1 (26.0, 28.8)	0.5	26.5 (25.4, 28.4)	27.4 (25.5, 28.9)	0.2
Unknown	3	1		3	1	
Inter foramen distance	46.8 (44.3, 49.2)	47.2 (45.0, 49.3)	0.4	46.8 (44.3, 49.2)	47.2 (45.0, 49.3)	0.4
Unknown	4	3		4	3	
Length of anterior loop for type III	2.40 (1.83, 3.00)	2.90 (2.00, 3.90)	0.034	2.20 (1.50, 2.98)	2.30 (1.80, 3.10)	0.3

**Figure 2:**
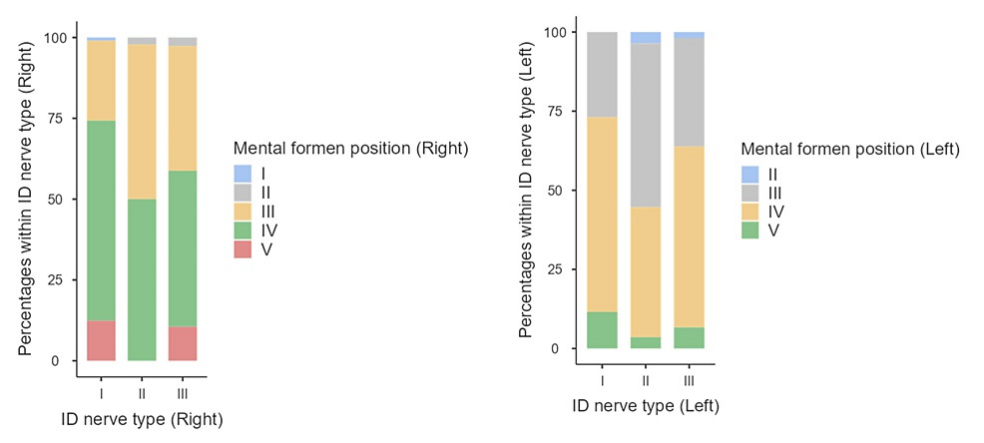
Association between the ID nerve type and mental foramen positions. ID: Inferior dental

There was a significant association between the inferior dental nerve and the position of the MF on both sides as shown in Table 3. On the right side, 65 (62%) of patients with type I inferior dental had a MF laying in position IV, while 22 (48%) of patients with type II inferior dental nerve had a MF laying in position III (p-value=0.004). On the left side, 64 (62%) of patients with type I inferior dental nerve had a MF laying in position IV, while 29 (52%) of patients with type II inferior dental nerve had a MF laying in position III as shown in Figure 2.

**Table 3:** Association between the ID nerve type and mental foramen positions. ^1^n (%) ^2^Fisher’s exact test

Characteristic	Right-side				Left-side			
	I (N = 107^1)^	II (N = 46^1^)	III (N = 115^1^)	p-value^2^	I (N = 105^1^)	II (N = 58^1^)	III (N = 105^1^)	p-value^2^
Mental foramen position				0.004				0.010
I	1 (1.0%)	0 (0%)	0 (0%)		-	-	-	
II	0 (0%)	1 (2.2%)	3 (2.6%)		0 (0%)	2 (3.6%)	2 (1.9%)	
III	26 (25%)	22 (48%)	44 (39%)		28 (27%)	29 (52%)	36 (34%)	
IV	65 (62%)	23 (50%)	55 (48%)		64 (62%)	23 (41%)	60 (57%)	
V	13 (12%)	0 (0%)	12 (11%)		12 (12%)	2 (3.6%)	7 (6.7%)	

There was a significant association between the midline-mental foramen distance and the position of the MF on the right side, in which patients with mental foramen laying in position V had a significantly higher midline-mental foramen distance, with a median of 28.6 mm (IQR: 26.4, 29.4) (p-value=0.003) as shown in Table 4. Additionally, there was a significant association between mental foramina laying in position IV and higher inter-foramen distance on the right side, with a median of 47.4 mm (IQR: 45.5, 49.8) (p-value=0.028). On the left side, there was a significant association between mental foramina laying in position IV and higher midline-mental foramen distance, with a median of 27.5 mm (IQR: 25.7, 29.2) (p-value=0.014), and higher inter-foramen distance in mental foramina laying in positions IV, with a median of 47.3 mm (IQR: 45.2, 49.9) (p-value=0.020).

**Table 4:** Anatomical measurements based on mental foramen positions. ^1^Median (IQR) ^2^Kruskal-Wallis rank sum test

Characteristic	II (N = 4^1^)	III (N = 92^1^)	IV (N = 143^1^)	V (N = 25^1^)	p-value^2 ^
	Right-sided				
Midline-mental foramen distance	25.5 (24.6, 26.7)	26.6 (25.25, 28.0)	27.4 (26.2, 28.8)	28.6 (26.4, 29.4)	0.003
Inter foramen distance	45.2 (42.9, 47.2)	46.4 (43.4, 48.8)	47.4 (45.5, 49.8)	46.9 (44.6, 49.6)	0.028
Length of anterior loop for type III	2.50 (2.45, 2.50)	2.30 (1.78, 3.20)	2.80 (2.00, 3.80)	3.30 (2.53, 4.23)	0.2
	Left-sided				
Midline-mental foramen distance	24.9 (23.9, 25.9)	26.5 (24.9, 27.9)	27.5 (25.7, 29.2)	27.2 (25.5, 28.6)	0.014
Inter foramen distance	44.5 (42.9, 45.9)	46.4 (43.7, 48.7)	47.3 (45.2, 49.9)	47.3 (45.0, 50.0)	0.020
Length of anterior loop for type III	2.80 (2.50, 3.10)	2.10 (1.65, 2.53)	2.30 (1.50, 3.20)	3.30 (2.63, 3.83)	0.2

There was a significant association between the type of inferior dental nerve and midline-mental foremen distances on both sides. Specifically, on the right and left sides, patients with type I inferior dental nerve had a significantly higher midline-mental foramen distance, with median values of 27.65 mm (IQR: 26.40, 28.80) on the right side and 27.60 mm (IQR: 26.20, 29.03) on the left side (p-values of 0.008 and 0.004, respectively), as shown in Table 5.

**Table 5:** Anatomical measurements based on the ID nerve type. ^1^Median (IQR) ^2^Kruskal-Wallis rank sum test ID: Inferior dental

Characteristic	I (N = 107^1^)	II (N = 46^1^)	III (N = 115^1^)	p-value^2 ^
	Right-sided			
Midline-mental foramen distance	27.65 (26.40, 28.80)	26.40 (25.20, 28.10)	26.80 (25.70, 28.30)	0.008
Inter foramen distance	47.5 (45.4, 50.0)	45.8 (44.0, 49.2)	46.6 (44.2, 48.8)	0.091
	Left-sided			
Midline-mental foramen distance	27.60 (26.20, 29.03)	25.95 (24.25, 27.80)	27.20 (25.50, 28.65)	0.004
Inter foramen distance	47.3 (45.5, 49.3)	45.4 (43.5, 49.0)	47.1 (45.0, 49.2)	0.054

## Discussion

The evaluation of the loop of the nerve holds great importance in dental practice as it ensures the safety and effectiveness of surgical procedures in the mandibular region. In our research, we utilized CBCT scans to evaluate the variations in MF types and AL characteristics in a sample of Jordanian individuals.

One of the most important discoveries of the research is the diversity of foramen types within the population. We observed classifications of foramen types, specifically highlighting Type III mental foramen, which exhibited a nerve loop; other similar studies also found these findings in different populations [13]. This classification has been extensively studied in the literature, and it has significant implications, in the field of dentistry. It is crucial to understand these variations to plan treatments and enhance care [14]. By identifying the types of MF, AL dental professionals can be better equipped to handle potential anatomical differences and minimize complications during surgical procedures.

Type III was the most prevalent in our sample, both in males and in females. Except, females on the left side had type I and type III equally prevalent. This was in contrast to a Saudi study that found that type I was the most prevalent [15]. One of the main findings of our study is the difference in the length of the AL for type III regarding gender, in which males had a slightly higher length (median difference = 0.5 mm), highlighting the importance of considering gender-specific variations in treatment planning. Whether this finding is consistent with the literature depends on the population being studied. Barbosa et al. have found no difference in general between males and females in their meta-analysis [16]. However, included studies have found that males have a greater length in some populations, including the Taiwanese population [17].

In our study, we also focused on measuring the length of the AL; for Type III on both sides, left and right the average lengths found were 2.9 mm and 2.69 mm, respectively. However, it is important to note that the recorded lengths varied widely, ranging from 1 mm to 8 mm, this indicates a diversity in the loop length among individuals. It emphasizes the importance of interpreting CBCT scans and tailoring treatment plans to each individual to prevent any nerve injuries during critical dental procedures like implants and flap elevation. In addition, we explored the connection between how the MF is classified and the type of nerve it corresponds to [18]. Our analysis unveiled a link between these two factors, indicating that the classification of the nerve can be influenced by the MF [19].

This discovery underscores the significance of considering the classification of the foramen, during treatment planning [20]. By understanding this relationship, dental professionals can anticipate if an AL is present and adjust their approach accordingly to minimize any harm to nerves.

There were some discrepancies between two- and three-dimensional imaging. Cone beam measurements of the mental foramina and the MF showed larger average separations from the midline and from each other than did PAN measurements. For AL lengths, however, it was the opposite. CBCT gets beyond some of the drawbacks of two-dimensional imaging, including superimposition, magnification, and distortion. It has been demonstrated that CBCT-reformatted panoramic pictures, which are devoid of superimposition and magnification, are more effective than PAN at identifying the mandibular canal [21].

The findings of this study are important for professionals working with the Jordanian population. By comprehending the prevalence of MF types and variations in the length of the AL of the MN, dental practitioners can enhance their treatment approaches and deliver improved care to patients. This knowledge can help minimize the chances of nerve damage during procedures and ultimately lead to better patient outcomes [21].

However, this study also has several limitations. Firstly, the retrospective nature of our study raises concerns about recall and selection bias, this can introduce inaccuracies and compromise the reliability of the collected data. Second, the sample size is small in our study which may reduce the generalizability of the results. Also, operator bias in interpreting CBCT scans is another concern, as variations in individual skill can affect the accuracy of assessments regarding the AL of the MN. This is a limitation due to a lack of reliable data and intra- and interobserver calibration. Thus, conducting larger long-term prospective studies can enhance the reliability and generalizability of study findings, providing a more comprehensive understanding of the clinical outcomes and potential complications associated with the presence of an AL during dental procedures.

## Conclusions

This study highlights the significance of utilizing CBCT scans to evaluate the AL within the Jordanian population. Through analyzing CBCT images and measuring the type and length of AL, valuable insights have been gained regarding variations and their clinical implications. The research findings highlight the importance of understanding the AL to ensure safe and effective dental procedures and the correlation between the AL length and the types of mental foramina. By establishing protocols and minimizing complications, this knowledge can greatly benefit patients through optimized treatment planning and improved patient care. Moreover, CBCT technology has proven invaluable in providing images of facial structures, enabling dentists to accurately assess and evaluate the AL. This advanced imaging technique significantly enhances care quality while contributing to advancements in dentistry.
